# Medication overdose data analysis: a review of medication error reports in the FDA adverse event reporting system (FAERS)

**DOI:** 10.1186/s40360-023-00681-y

**Published:** 2023-08-04

**Authors:** Jiaqi Ni, Xinru Tang, Li Chen

**Affiliations:** 1 Department of Pharmacy/Evidence-Based Pharmacy Center, West China Second University Hospital, Sichuan University, Chengdu, China; 2 Key Laboratory of Birth Defects and Related Diseases of Women and Children, Sichuan University, Ministry of Education, Chengdu, China; 3 West China School of Pharmacy, Sichuan University, Chengdu, China

**Keywords:** Drug safety, Drug overdose, Medication errors, FAERS, Patient safety

## Abstract

**Background:**

drug overdose is a common type of medication error, which caused significant patient injuries and economic losses. To determine which drugs are reported most frequently in association with drug overdose, a comprehensive search was conducted in the FDA Adverse Event Reporting System (FAERS) database. The study also sought to determine the top 10 drugs reported with drug overdose.

**Methods:**

FAERS database was searched for drug overdose records submitted from the first quarter of 2017 to the fourth quarter of 2021. Descriptive analyses were conducted based on the total counts and percentages of reports associated with the drug. Subgroup analyses were performed on drugs of different pharmacological classifications.

**Results:**

A total of 170,424 drug overdose reports were retrieved. The results revealed that antipyretics and analgesics took the highest risk for overdose, with 63,143 (37.05%) cases reported. Among them, opioids were associated with the most drug overdose events. The top 10 drug classes relating to drug overdose in FAERS were opioid analgesic, anilide antipyretic analgesic, 5-HT reuptake inhibitors, bronchodilators, monoclonal antibodies and antibody-drug conjugates, benzodiazepines, antipsychotics, GABA derivatives, antimanic agents, and propionic acid derivatives.

**Conclusion:**

to reduce the occurrence of drug overdose events, some methods could be considered including applying a pre-prescription review system, drug safety education, developing warning lists, etc.

**Supplementary Information:**

The online version contains supplementary material available at 10.1186/s40360-023-00681-y.

## Key Points

Antipyretic analgesic and nervous system drugs were reported with the highest overdose risks. The top 3 drug classes with overdose risks were opioid analgesic, anilide, and 5-HT reuptake inhibitors.

**Supplementary Information:**

The online version contains supplementary material available at 10.1186/s40360-023-00681-y.

## Introduction

Patient safety is a public health concern globally, of which drug safety is an essential part. The World Health Organization (WHO) released the “global patient safety challenge - drug safety” in Bonn, Germany in March 2017, aiming to decrease serious and avoidable drug-related injuries in all countries by 50% in the next five years [1]. Medication error (ME) refers to any malpractice in the medication treatment process which may occur during all stages of medication use from ordering, through prescribing, dispensing, and administration, to monitoring [2, 3]. MEs constitute a major challenge to patient safety and are associated with patient harm and remarkable national expenses. Consequently, healthcare authorities in many countries have adopted patient safety reporting systems to routinely collect data on MEs [2]. Drug overdose is a common type of ME, which remains a serious health concern worldwide. Data from the National Vital Statistics System (NVSS) reported 91,799 drug overdose deaths in the United States in 2020 [4]. The rate of drug overdose deaths increased from 2019 to 2020 among all races, Hispanic-origin groups, and groups aged 15 and over [4]. In Japan, drug overdose was the major cause of emergency room visits and the leading cause of tertiary hospital admissions [5]. The direct medical costs of drug overdose were estimated to be 7.7 billion yen per year [6].

The Food and Drug Administration (FDA) Adverse Event Reporting System (FAERS) is a database designed to support FDA’s post-marketing safety surveillance program for drugs and therapeutic biological products [7]. It contains adverse event reports FDA has received from drug manufacturers as required by regulations along with reports submitted by patients and health care providers [8]. Adverse event reports, medication error reports, and product quality complaints resulting in adverse events were submitted spontaneously to improve drug safety [9]. With a large amount of data and free access to the public, the FAERS database was widely used in pharmacovigilance analyses [10].

To the best of our knowledge, no studies have been conducted with the FAERS database to determine which drugs are reported most frequently in association with drug overdose. This study aimed to evaluate the scope to which specific drugs are reported relating to drug overdose. We also sought to determine the top 10 drugs reported with drug overdose in the FAERS database.

## Methods

### Data source

A comprehensive search of drug overdose records was performed with the publicly available FAERS database on August 8th, 2022. Reports submitted from the first quarter of 2017 to the fourth quarter of 2021 (January 1st, 2017 to December 31st, 2021) were retrieved. Anonymized data including patient demographic information (DEMO file), drug information (DRUG file), and adverse reaction information (REAC file) were collected. Records with the Preferred Terms (PTs) containing “overdose” in the REAC file and associated patient demographic and drug information were extracted for further analysis. Authors had no access to information that could identify individual participants during or after data collection.

### Data mining

Navicat Premium (version 11.0.8) and MySQL (version 5.7) were adopted to deal with the data cleaning and normalization process, including merging data, deleting duplicated records, applying standardized vocabulary, and standardizing the response to Medical Dictionary for Regulatory Activities (MedDRA, version 25.0) concept. Reaction information was standardized to the PTs including “overdose”, “intentional overdose”, “accidental overdose”, and “prescribed overdose”. In total, 170,424 drug overdose reports were retrieved.

### Statistical analysis

To assess the scope to which specific drugs are reported in association with drug overdose, descriptive analyses were conducted based on the total counts and percentages of reports associated with the drug. Subgroup analyses were performed on drugs of different pharmacological classifications to determine those with higher risks for overdose.

## Results

### General information

A total of 170,424 drug overdose reports were retrieved retrospectively. Among them, 39,736 (23.32%) cases were reported as intentional overdoses. 13,028 (7.64%) were reported as accidental overdoses. 10,074 (5.91%) were reported as prescribed overdoses. The rest of the cases were reported as overdoses. In addition, adults (aged 18–60 years old) and the elderly (aged over 60 years old) were reported with 50,929 (29.88%) and 24,961 (14.65%) drug overdose events, respectively. In terms of gender, 39.64% of drug overdose events occurred in males, 46.61% occurred in females, and 13.75% were reported with unknown gender. Regarding the route of administration, drug overdose tended to occur more frequently in the oral route, which accounted for 49.68% of reports. 55,728 (32.7%) cases were reported with unknown routes of administration. Furthermore, sustained and controlled-release tablets took high risks for a drug overdose, with 33,232 (19.50%) cases reported, followed by conventional tablets. The clinical characteristics of drug overdose events were demonstrated in Table 1.

**Table 1:** Clinical characteristics of drug overdose events collected from the FAERS database

Characteristics	Number of reports (N = 170,424)	Percentage (%)
*Age group*		
Newborn (0 to 28 d)	127	0.07
Infant (29 d to 3 y)	1,878	1.10
Child (3 to 12 y)	2,338	1.37
Teenager (12 to 18 y)	10,076	5.91
Adult (18 to 60 y)	50,929	29.88
Elderly (over 60 y)	24,961	14.65
Unknown	80,115	47.01
*Gender*		
Male	67,548	39.64
Female	79,438	46.61
Unknown	23,438	13.75
*Route of administration*		
Oral	84,666	49.68
Transdermal	13,753	8.07
Intravenous	5,472	3.21
Subcutaneous	4,766	2.80
Through respiratory tract	2,870	1.68
Intramuscular	1,301	0.76
Others	1,868	1.10
Unknown	55,728	32.70
*Dosage form*		
Tablet		
Conventional tablet	32,636	19.15
Sustained/controlled release tablet	33,232	19.50
Coated tablet	5,042	2.96
Dispersible tablet	525	0.31
Chewable tablet	283	0.17
Enteric-coated tablet	138	0.08
Other tablets	131	0.08
Injection	14,361	8.43
Gel	10,131	5.94
Capsule	8,110	4.76
Inhaler	5,041	2.96
External dosage form		
Transdermal patch	2,215	1.30
Paint	1,435	0.84
Drop	512	0.30
Other external dosage forms	242	0.14
Others	3,121	1.83
Unknown	53,269	31.26

### Drug overdose in drugs of different pharmacological classifications

Subgroup analyses were conducted on drugs of different drug classes. Suspected drugs were classified into 14 groups, as demonstrated in Table 2. The results revealed that antipyretics and analgesics took the highest risk for overdose, with 63,143 (37.05%) cases reported. 37,402 (21.95%) cases related to nervous system drugs were reported, indicating a high frequency of drug overdose. For each drug category, drugs were further analyzed based on the total counts and percentages of associated reports.

**Table 2:** Drug categories of suspected drugs for drug overdose events

Drug category	Number of reports (n = 170,424)	Percentage (%)
Antipyretic and analgesic	63,143	37.05
Nervous system drug	37,402	21.95
Antineoplastic	9,532	5.59
Respiratory drug	8,707	5.11
Cardiovascular drug	8,116	4.76
Endocrine drug	6,296	3.69
Immune drug	4,541	2.66
Digestive system drug	3,699	2.17
Blood system drug	3,536	2.07
Autacoid	2,876	1.69
Anti-infective drug	2,821	1.66
Reproductive system drug	1,834	1.08
Anesthetic	1,025	0.60
Others	16,483	9.67
Unknown	413	0.24

#### Drug overdose in antipyretics and analgesics

63,143 drug overdose events were reported in association with antipyretic and analgesic use. Relevant drugs included opioids, propionic acid derivatives, salicylic acids, COX-2 inhibitors, and acetic acid derivatives, etc. Among them, opioids accounted for 81.90%, with 51,715 cases reported. Morphine, oxycodone, hydrocodone, buprenorphine, codeine, meperidine, and tramadol were representative drugs (Table S1).

#### Drug overdose in the nervous system drugs

37,402 events were reported in relationship with drugs acting on the nervous system. Suspecting drugs included antidepressants, antipsychotics, anti-Parkinson agents, antiepileptic agents, antimanic drugs, and sedatives, etc. Among them, 5-HT reuptake inhibitors, benzodiazepines, and antipsychotics were associated with higher overdose risks (Table S1).

#### Drug overdose in antineoplastic agents

Antineoplastic agents were suspected to cause 9,532 drug overdose events, including monoclonal antibodies, tyrosine kinase inhibitors, hormone antagonists, plant alkaloids, etc. Among them, monoclonal antibodies and antibody-drug conjugates were associated with higher risks, with 5,155 (54.08%) cases reported. Ipilimumab, rituximab, and nivolumab were representative drugs (Table S1).

#### Drug overdose in respiratory drugs

Drugs acting on the respiratory system were suspected to result in 8,707 drug overdose events. Suspecting drugs included bronchodilators, corticosteroids, cough suppressants, etc. Bronchodilators, including salbutamol, terbutaline, and salmeterol were associated with higher risks of overdose, with 5,609 (64.42%) cases reported (Table S1).

#### Drug overdose in cardiovascular drugs

Cardiovascular drugs such as calcium channel blockers, antiarrhythmic drugs, and vasodilators were related to 8,116 events. Calcium channel blockers, including amlodipine and nifedipine, were associated with higher risks, with 2,957 (36.43%) cases reported (Table S1).

#### Drug overdose in endocrine drugs

6,296 drug overdose events were reported in association with endocrine drugs. Related drugs included anti-diabetic, hormone, anti-gout agents, etc. Biguanide, or more specifically, metformin accounted for 38.17%, with 2,403 cases reported (Table S1).

#### Drug overdose in immune drugs

4,541 events were reported in a relationship with immune drugs. Suspecting drugs included JAK inhibitors, TNF inhibitors, anti-inflammatory drugs, immunosuppressants, etc. Among them, TNF inhibitors such as etanercept and adalimumab were associated with higher overdose risks, accounting for 38.05% of the events (Table S1).

#### Drug overdose in digestive system drugs

Drugs acting on the digestive system were suspected to cause 3,699 drug overdose events, including antacids, H_2_ receptor antagonists, proton pump inhibitors (PPIs), antiemetics, laxatives, etc. Among them, PPIs, such as omeprazole and lansoprazole were associated with higher risks, with 1,288 (34.82%) cases reported (Table S1).

#### Drug overdose in blood system drugs

Drugs acting on the blood system were suspected to result in 3,536 drug overdose events. Suspecting drugs included anti-anemic, antiplatelet agents, anticoagulants, and platelet stimulating agents, etc. Anticoagulants, including heparin, warfarin, and apixaban were associated with higher risks of overdose, with 2,403 (67.96%) cases reported (Table S1).

#### Drug overdose in autacoids

Autacoids including H_1_ receptor antagonists and 5-HT receptor agonists were related to 2,876 events. H_1_ receptor antagonists, with cetirizine and diphenhydramine as representative drugs were associated with higher risks. 2,700 (93.88%) drug overdose events were reported (Table S1).

#### Drug overdose in anti-infective drugs

2,821 drug overdose events were reported in association with anti-infective drug use. Relevant drugs included antibiotics, antifungal agents, anti-HIV agents, etc. Anti-HIV agents, lamivudine, and emtricitabine accounted for 17.16%, with 484 cases reported (Table S1).

#### Drug overdose in reproductive system drugs

1,834 events were reported in relationship with drugs acting on the reproductive system. Suspecting drugs included estrogen, antiestrogen, androgen, antiandrogen, etc. Among them, male reproductive systems drugs such as tadalafil and sildenafil were more likely to be overused, accounting for 43.68% of the events (Table S1).

#### Drug overdose in anesthetic drugs

Anesthetic drugs were suspected to cause 1,025 overdose events. Intravenous general anesthesia such as chloriodarone and propofol was associated with higher risks, with 679 (66.24%) cases reported (Table S1).

### The top 10 drug classes associated with drug overdose

**Fig. 1:**
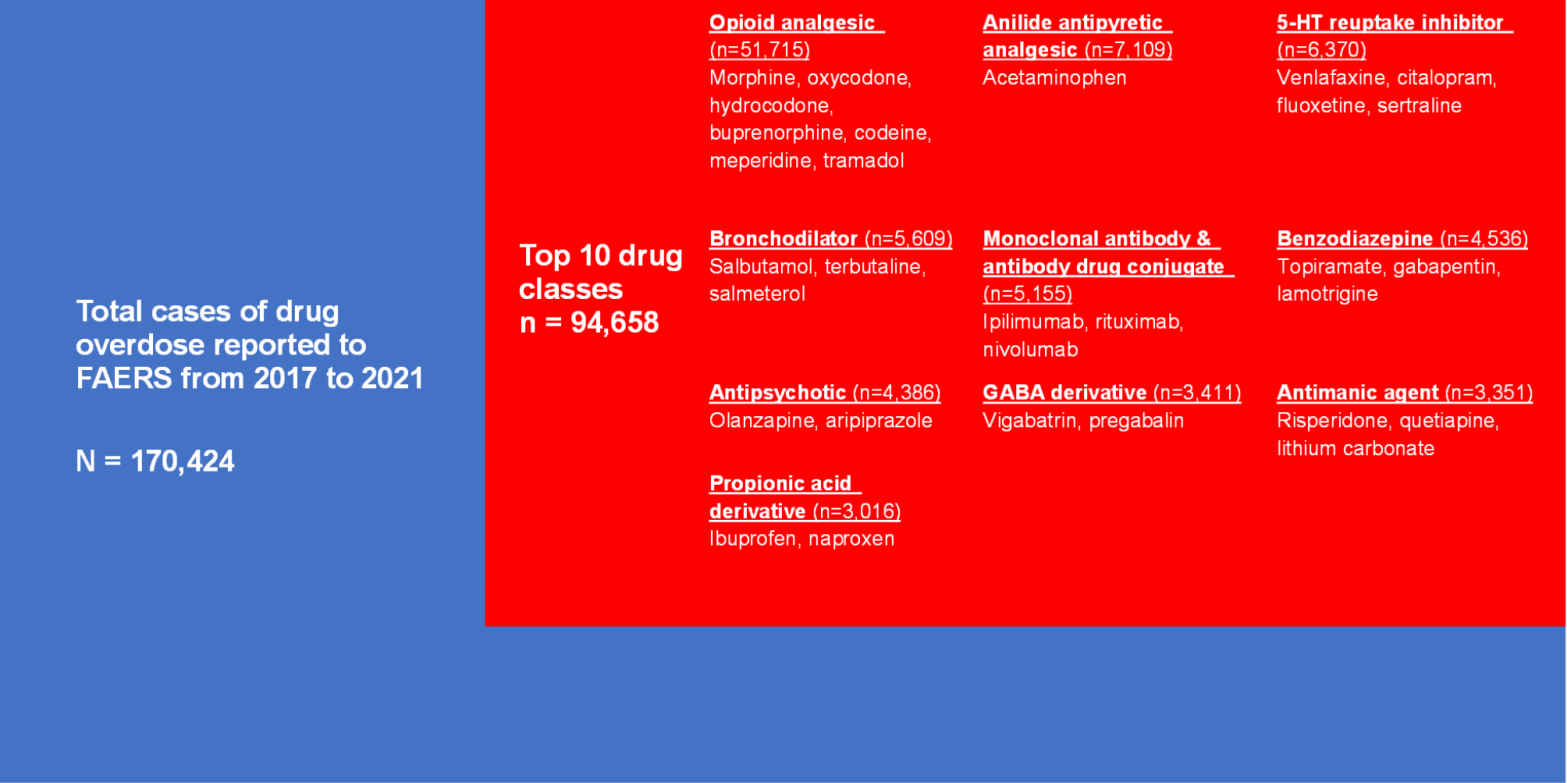
Top 10 drug classes associated with drug overdose reported to FAERS from 2017 to 2021. The relative size of rectangles correlates with the percent of total cases reported

From the first quarter of 2017 to the fourth quarter of 2021, a total of 170,424 drug overdose events were reported to the FDA. The top 10 drug classes associated with drug overdose were shown in Fig. 1. These top 10 drug classes comprised 55.54% (94,658) of all events reported. Opioid analgesic was the most reported drug class in association with overdose, comprising 30.34% (51,715) of all reports. Anilide antipyretic analgesic was the second class of drug relating to overdose, with 4.17% (7,109) of all reports, followed by 5-HT reuptake inhibitors (3.74%) and bronchodilators (3.29%). The top 10 drug classes reported to the FDA relevant to drug overdose were opioid analgesic, anilide antipyretic analgesic, 5-HT reuptake inhibitors, bronchodilators, monoclonal antibodies and antibody-drug conjugates, benzodiazepines, antipsychotics, GABA derivatives, antimanic agents, and propionic acid derivatives in descending order.

## Discussion

### Drug overdose in elderly patients

Drug overdose events reported in elderly patients (age over 60 years old) accounted for 14.65% (24,961/170,424) of all cases. Elderly patients were vulnerable to drug overdose. Firstly, the functions of hearts, livers, kidneys, and other organs declined as patients get older. The pharmacokinetic processes including the absorption, distribution, metabolism, and excretion of drugs in the body changed remarkably as a result. Drug dosage for adults might have overdosed for elderly patients. Secondly, with the decline of physical function, elderly patients took higher risks of developing chronic diseases, such as hypertension, diabetes, hyperlipidemia, coronary artery diseases, etc. Elderly patients often suffer from multiple co-morbidities and have to take multiple drugs at the same time. They were more likely to forget whether they have taken drugs and chose to take drugs again because of poor memory, which made them more likely to overdose when taking drugs. Thirdly, the decline in cognitive function was common in elderly patients, which brought difficulty appraising health information. A cross-sectional survey including 440 participants reported low health literacy levels among the elderly [11]. Moreover, elderly patients tend to have poor medication compliance, which was manifested by withdrawing drugs on their own, polypharmacy shopping without doctor’s diagnoses or prescriptions, and increasing doses when they held the view that the drug was not working [12].

### Drug overdose of oral medications

Oral medications accounted for 49.68% (94,666/170,424) of all reported drug overdose events. Sustained/controlled-release tablets were more likely to overdose compared to drugs in other dosage forms. Oral administration of drugs was a convenient, and non-invasive route to take medications. Additionally, the oral route was an acceptable way for self-administration of medications. Without guidance and supervision from health care providers, patients were more likely to overdose themselves. Sustained/controlled-release tablets usually contain high doses of medications, which were released gradually in the body. If patients take them more frequently than normal, serious drug overdose events could occur.

### Drug overdose of antipyretics and analgesics

Drug overdose occurred most frequently in antipyretics and analgesics, which accounted for 37.05% (63,143/170,424) of all reported cases. Among antipyretics and analgesics, opioids were most likely to cause drug overdose, occupying 81.90% (51,715/63,143). In addition, opioids ranked first in the top 10 drug classes associated with a drug overdose. Opioids include opioid alkaloid analgesics, synthetic analgesics, and some endogenous opioid peptides. Opioids are widely used in clinical settings with their powerful analgesic effects. They mainly exert their pharmacological effects by combining with opioid receptors. Because of the addictive nature of opioids, patients may take excessive drugs unconsciously with the urging of drug addiction and even cause life-threatening outcomes. Opioid addiction and overdose deaths have continually devastated countries such as the United States and Canada [13]. In the past two decades, nearly 600,000 people in these two countries died from opioid overdose events. Moreover, it was estimated that 1.2 million people might die from opioid overdose by 2029 [14].

### Methods to reduce drug overdose events

Some methods could be adopted to reduce drug overdose events and improve drug safety. Tan et al. reported the implementation of an electronic pre-prescription system to detect prescription overdoses [15]. All prescriptions were automatically reviewed by the system. Then any potentially inappropriate prescriptions were reviewed by pharmacists before dispensing and payment. Pharmacists updated review rules of prescriptions in the pre-prescription system to improve efficiency and accuracy. The pre-prescription review system served as an effective tool to reduce prescription overdoses. To reduce prescription overdoses, pharmacists should update the dose range of drugs in the system routinely [16]. Another method is to develop warning lists for drugs with high overdose risks. Each institution could establish its lists based on reports of drug overdose events. The list could also be established based on the top 10 drug classes identified by our research. The warning drug lists should be updated promptly. Similarly, some modifications could be made to the current electronic drug ordering system. When drugs on the warning lists were ordered, a Best Practice Advisory box would pump up with dose recommendations, to avoid prescription overdoses. Apart from doctors’ prescriptions, nurses’ implementation of drug orders, pharmacists’ reading of prescriptions, and sometimes patients themselves and their families play a role in drug overdoses [3]. Among them, pharmacists play a major role in detecting drug overdoses in the process of reviewing prescriptions, dispensing drugs, conducting patient education, and communicating with medical teams. When dispensing drugs, overdose prescriptions could be sent back to physicians for correction. The role of the pharmacist in preventing polypharmacy has received greater attention. Questioning the prescription and providing information to prescribing physicians may be effective in reducing polypharmacy and reducing intentional overdoses [17]. When educating patients, special attention could be paid to improving patients’ health literacy and adherence to prescriptions, aiming to reduce accidental and intentional overdoses. For elderly patients, patient education combined with alarm clocks as reminders would help to reduce drug overdose events. Moreover, some lectures or activities on drug safety could be carried out in the community to improve patients’ health literacy. For health institutions, when drug overdose events occur, institutions should conduct a related investigation to figure out the causes of events. Methods should be applied to improve the health system and avoid the occurrence of events. Related education and training should be communicated to personnel promptly.

### Limitations and prospects

Some limitations were identified for our research. Firstly, as FAERS is a spontaneous reporting system, overdose event reports collected might be incomplete or duplicated, with the possibility of missing or false reporting. Significant biases in reporting can exist based on national attention or regional awareness. For example, 47.01% of cases were reported with unknown age, and 32.7% of cases were reported with unknown route of administration in our study. Secondly, all drugs given in association with adverse drug reactions were reported to FAERS. Firm causality of an individual drug related to the adverse event could be difficult to determine [9, 18]. The event may have been related to the underlying disease being treated, caused by some other drugs being taken concurrently, or occurred for other reasons. The information in FAERS reports reflects only the reporter’s observations and opinions. Thirdly, our study found that the oral route of administration and elderly age as important factors in overdose cases. We were not surprised at the results as oral medications were most commonly prescribed. In addition, some drugs were reported with more overdose events in the elderly age group as they were prescribed more often in elderly populations, which indicated another limitation of FAERS, the lack of an unbiased denominator to enable robust comparisons. In addition, drug overdose events in the FAERS database were mainly reported in the United States and some European countries. The results did not apply to other regions with different populations.

FAERS is a publicly available post-marketing safety surveillance database with millions of real-world spontaneous drug safety reports. The large quantity of data collected from a large population around the world makes FAERS robust for conducting pharmacovigilance studies in real-world settings [19]. FAERS is intended to be used as an early-warning signal detection system. It can be used to generate hypotheses regarding potential drug safety issues that either weren’t identified during clinical trials or emerged over time due to other factors. Our study was designed to determine the drugs most frequently reported with drug overdose in the FAERS database. However, due to the limitations of FAERS itself, rigorous post-marketing studies and further investigations with less biased, more complete datasets, such as healthcare claims or electronic health records (EHRs) were required to establish reliable drug-event associations.

## Conclusions

Our research comprehensively analyzed the drug overdose event reports in the FAERS database from 2017 to 2021, aiming at raising concerns for drug safety. Antipyretic analgesic and nervous system drugs were the categories of drugs with the highest overdose risks. The top 3 drug classes associated with overdose were opioid analgesic, anilide with analgesic and antipyretic activity, and 5-HT reuptake inhibitors. To reduce the occurrence of drug overdose events, some methods could be considered including applying a pre-prescription review system, pharmacists’ involvement in interrupting drug overdose events, drug safety education to the public and health care personnel, and developing warning lists for drugs with high overdose risks, etc.

### Electronic supplementary material

Below is the link to the electronic supplementary material.

Supplementary Material 1

Supplementary Material 2

## Data Availability

The dataset used during the current study is available in the supplementary section (Supplementary File S2).
