# Supplementary material for: Medication overdose data analysis: a review of medication error reports in the FDA adverse event reporting system (FAERS)
Source: BMC Pharmacol Toxicol. 2023 Aug 4;24:41. doi: 10.1186/s40360-023-00681-y (PMC10403938; doi:10.1186/s40360-023-00681-y)
Supplement: Supplementary file 1 — Supplementary Material 1 [file 40360_2023_681_MOESM1_ESM.docx]

Table S1. Drug overdose in drugs of different drug classes

| **Antipyretic and analgesic** | | | |
| --- | --- | --- | --- |
| *Drug class* | *Number of reports*  *(n=63,143)* | *Percentage (%)* | *Representative drugs* |
| Opioid | 51,715 | 81.90 | Morphine, oxycodone, hydrocodone, buprenorphine, codeine, meperidine, tramadol |
| Anilide | 7,109 | 11.26 | Acetaminophen |
| Propionic acid derivative | 3,016 | 4.78 | Ibuprofen, naproxen |
| Salicylic acid | 617 | 0.98 | Aspirin |
| Acetic acid derivative & related substance | 547 | 0.87 | Diclofenac |
| COX-2 inhibitor | 91 | 0.14 | Celecoxib |
| Others | 48 | 0.08 | Meloxicam |
| **Nervous system drug** | | | |
| *Drug class* | *Number of reports*  *(n=37,402)* | *Percentage (%)* | *Representative drugs* |
| Tricyclic antidepressant | 104 | 0.28 | Doxepin, clomipramine |
| 5-HT reuptake inhibitor | 6,370 | 17.03 | Venlafaxine, citalopram |
| Other antidepressants | 2,616 | 6.99 | Bupropion, mirtazapine |
| Antipsychotic | 4,386 | 11.73 | Olanzapine, aripiprazole |
| Dopaminergic agent | 1,280 | 3.42 | Carbidopa |
| Other anti-Parkinson agents | 174 | 0.47 | Rasagiline, droxidopa |
| Hydantoin derivative | 223 | 0.60 | Phenytoin |
| Sodium channel blocker | 1,835 | 4.91 | Carbamazepine, lamotrigine |
| GABA derivative | 3,411 | 9.12 | Pregabalin |
| Other antiepileptics | 3,183 | 8.51 | Gabapentin, levetiracetam |
| Antimanic drug | 3,351 | 8.96 | Risperidone, quetiapine, lithium carbonate |
| Benzodiazepine | 4,536 | 12.13 | Lorazepam, alprazolam |
| Sedative hypnotic | 2,839 | 7.59 | Zolpidem |
| Psycho-analeptic agent | 889 | 2.38 | Methylphenidate, amphetamine |
| Cholinergic agent | 92 | 0.25 | Galantamine, ristigmine |
| Anticholinergic | 609 | 1.63 | Botox, atropine |
| Adrenergic agent | 209 | 0.56 | Norepinephrine |
| Others | 1,295 | 3.46 | Baclofen |
| **Antineoplastic** |  |  |  |
| *Drug class* | *Number of reports*  *(n=9,532)* | *Percentage (%)* | *Representative drugs* |
| Monoclonal antibody & antibody drug conjugate | 5,155 | 54.08 | Ipilimumab, rituximab, nivolumab |
| Tyrosine kinase inhibitor | 1,116 | 11.71 | Afatinib, erlotinib |
| Antimetabolite | 530 | 5.56 | Capecitabine, fluorouracil |
| Hormone antagonist & related agent | 372 | 3.90 | Octreotide, abiraterone |
| Agent affecting the structure & function of DNA | 213 | 2.23 | Carboplatin, irinotecan |
| PARP inhibitor | 156 | 1.66 | Olapali, nirapali |
| Serine-threonine protein kinase inhibitor | 89 | 0.93 | Everolimus |
| Plant alkaloids and other natural products | 60 | 0.63 | Paclitaxel, vincristine |
| Cytotoxic antibiotic and related substance | 19 | 0.20 | Doxorubicin |
| Others | 1,820 | 19.09 | Interferon, ixazomib |
| **Respiratory drug** | | | |
| *Drug class* | *Number of reports*  *(n=8,707)* | *Percentage (%)* | *Representative drugs* |
| Bronchodilator | 5,609 | 64.42 | Salbutamol, terbutaline, salmeterol |
| Corticosteroid | 979 | 11.24 | Fluticasone, budesonide |
| Leukotriene receptor antagonist | 171 | 1.96 | Montelukast |
| Cough suppressant | 701 | 8.05 | Dextromethorphan |
| Expectorant | 479 | 5.50 | Guaifenesin |
| Others | 768 | 8.82 | Pseudoephedrine |
| **Cardiovascular drug** | | | |
| *Drug class* | *Number of reports*  *(n=8,116)* | *Percentage (%)* | *Representative drugs* |
| Calcium channel blocker | 2,957 | 36.43 | Amlodipine, nifedipine |
| Sympathetic inhibitor | 1,775 | 21.87 | Propranolol, labetalol |
| Angiotensin receptor blocker | 617 | 7.60 | Losartan, valsartan |
| Angiotensin converting enzyme inhibitor | 462 | 5.69 | Captopril, ramipril |
| Antiarrhythmic drug | 459 | 5.66 | Amiodarone, dofetilide |
| HMG CoA reductase inhibitor | 392 | 4.83 | Atorvastatin, simvastatin |
| Diuretic | 343 | 4.23 | Furosemide, hydrochlorothiazide |
| Other lipid-regulating drugs | 205 | 2.53 | Niacin, fenofibrate |
| Vasodilator | 154 | 1.90 | Epoprostenol sodium |
| Cardiotonic agent | 117 | 1.44 | Digoxin |
| Others | 635 | 7.82 | Bosentan |
| **Endocrine drug** | | | |
| *Drug class* | *Number of reports*  *(n=6,296)* | *Percentage (%)* | *Representative drugs* |
| Biguanide | 2,403 | 38.17 | Metformin |
| Insulin and its analog | 841 | 13.36 | Insulin |
| Insulin secretagogue | 1,426 | 22.65 | Glyburide, liraglutide |
| Other anti-diabetics | 207 | 3.29 | Empagliflozin |
| Glucocorticoid | 289 | 4.59 | Methylprednisolone, dexamethasone |
| Pituitary hormone | 270 | 4.29 | Growth hormone |
| Thyroid medication | 554 | 8.80 | Levothyroxine |
| Anti-gout agent | 186 | 2.95 | Allopurinol, colchicine |
| Others | 120 | 1.91 | Betaine, mecashermin |
| **Immune drug** | | | |
| *Drug class* | *Number of reports*  *(n=4,541)* | *Percentage (%)* | *Representative drugs* |
| JAK inhibitor | 774 | 17.04 | Tofacitinib |
| TNF inhibitor | 1,728 | 38.05 | Etanercept, adalimumab |
| Other anti-inflammatory & antirheumatic drugs | 411 | 9.05 | Ixekizumab |
| Immunosuppressant | 1,167 | 25.70 | Tacrolimus |
| Others | 461 | 10.15 | Teriflunomide |
| **Digestive system drug** | | | |
| *Drug class* | *Number of reports*  *(n=3,699)* | *Percentage (%)* | *Representative drugs* |
| Antacid | 86 | 2.32 | Calcium carbonate, aluminum hydroxide |
| H_2_ receptor antagonist | 364 | 9.84 | Ranitidine, famotidine |
| Proton pump inhibitor | 1,288 | 34.82 | Omeprazole, lansoprazole |
| Antiemetic | 158 | 4.27 | Onstatine, metoclopramide |
| Laxative | 733 | 19.82 | Polyethylene glycol, bisacodyl |
| Antidiarrheal | 933 | 25.22 | Loperamide |
| Others | 137 | 3.70 | Veldezumab |
| **Blood system drug** | | | |
| *Drug class* | *Number of reports*  *(n=3,536)* | *Percentage (%)* | *Representative drugs* |
| Anti-anemic agent | 208 | 5.88 | Chalybeate, folic acid |
| Leukocyte-stimulating agent | 144 | 4.07 | Pegfilgrastim, filgrastim |
| Platelet stimulating agent | 21 | 0.59 | Avatrombopag, futatinib |
| Antiplatelet agent | 173 | 4.89 | Clopidogrel, ticagrelor |
| Coagulant | 328 | 9.28 | Tranexamic acid, desmopressin |
| Anticoagulant | 2,403 | 67.96 | Heparin, warfarin, apixaban |
| Blood product | 115 | 3.25 | Human albumin |
| Others | 144 | 4.07 | Eculizumab |
| **Autacoid** | | | |
| *Drug class* | *Number of reports*  *(n=2,876)* | *Percentage (%)* | *Representative drugs* |
| H_1_ receptor antagonist | 2,700 | 93.88 | Cetirizine, diphenhydramine |
| 5-HT receptor agonist | 176 | 6.12 | Sumatriptan, buspirone |
| **Anti-infective drug** |  |  |  |
| *Drug class* | *Number of reports*  *(n=2,821)* | *Percentage (%)* | *Representative drugs* |
| Penicillin | 143 | 5.07 | Penicillin, amoxicillin |
| Cephalosporin | 140 | 4.96 | Cefuroxime, ceftriaxone |
| Other β lactams | 43 | 1.52 | Meropenem |
| Aminoglycoside | 30 | 1.06 | Gentamicin, amikacin |
| Tetracycline | 55 | 1.95 | Tetracycline, doxycycline |
| Macrolide | 106 | 3.76 | Erythromycin, azithromycin |
| Glycopeptide | 161 | 5.71 | Vancomycin |
| Other antibiotics | 163 | 5.78 | Linezolid, daptomycin |
| Synthetic antibacterial agent | 260 | 9.22 | Sulfamethoxazole, levofloxacin |
| Antifungal agent | 324 | 11.49 | Voriconazole, caspofungin |
| Anti-herpes virus agent | 351 | 12.44 | Acyclovir, valacyclovir |
| Anti-influenza | 90 | 3.19 | Amantadine, oseltamivir |
| Anti-HIV drug | 484 | 17.16 | Lamivudine, emtricitabine |
| Antimycobacterial | 97 | 3.44 | Rifampicin, isoniazid |
| Antiparasitic agent | 374 | 13.26 | Metronidazole, quinine |
| **Reproductive system drug** | | | |
| *Drug class* | *Number of reports*  *(n=1,834)* | *Percentage (%)* | *Representative drugs* |
| Androgen & antiandrogen | 230 | 12.54 | Testosterone, apalutamide |
| Other male reproductive system drugs | 801 | 43.68 | Tadalafil, sildenafil |
| Estrogen & antiestrogen | 199 | 10.85 | Estradiol, toremifene |
| Progestin & antiprogestin | 604 | 32.93 | Norethisterone, mifepristone |
| **Anesthetic** | | | |
| *Drug class* | *Number of reports*  *(n=1,025)* | *Percentage (%)* | *Representative drugs* |
| Inhaled general anesthesia | 10 | 0.98 | Sevoflurane |
| Intravenous general anesthesia | 679 | 66.24 | Chloriodarone, propofol |
| Local anesthetic | 336 | 32.78 | Lidocaine |
| COX-2: Cyclooxygenase-2; JAK: Janus kinase; TNF: Tumor necrosis factor | | | |
